# Supplementary material for: Identification and Construction of Strong Promoters in Yarrowia lipolytica Suitable for Glycerol-Based Bioprocesses
Source: Microorganisms. 2023 Apr 28;11(5):1152. doi: 10.3390/microorganisms11051152 (PMC10224437; doi:10.3390/microorganisms11051152)
Supplement: Supplementary file 1 [file microorganisms-11-01152-s001.zip › microorganisms-2369768-supplementary.pdf]

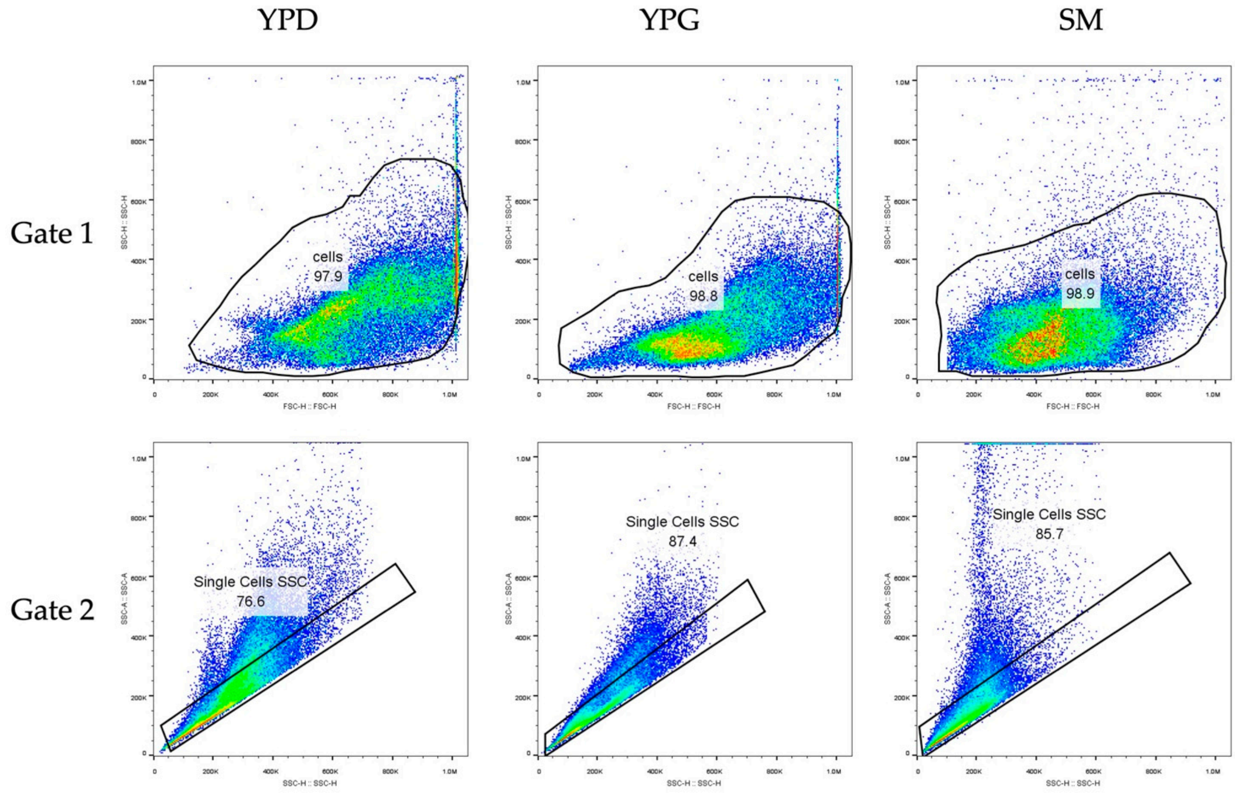

Figure S1. Example of the gating strategy used for the analysis of the flow cytometry data. The gates were implemented in the above order (Gate 1, Gate 2) in every sample. Gate 1 was implemented to exclude any debris in the sample, while Gate 2 was implemented to select only single cells and exclude any aggregates. The above subfigures depict the gates for one wild-type (WT 28849) biological replicate at 24 hours of culture in the three growth media.

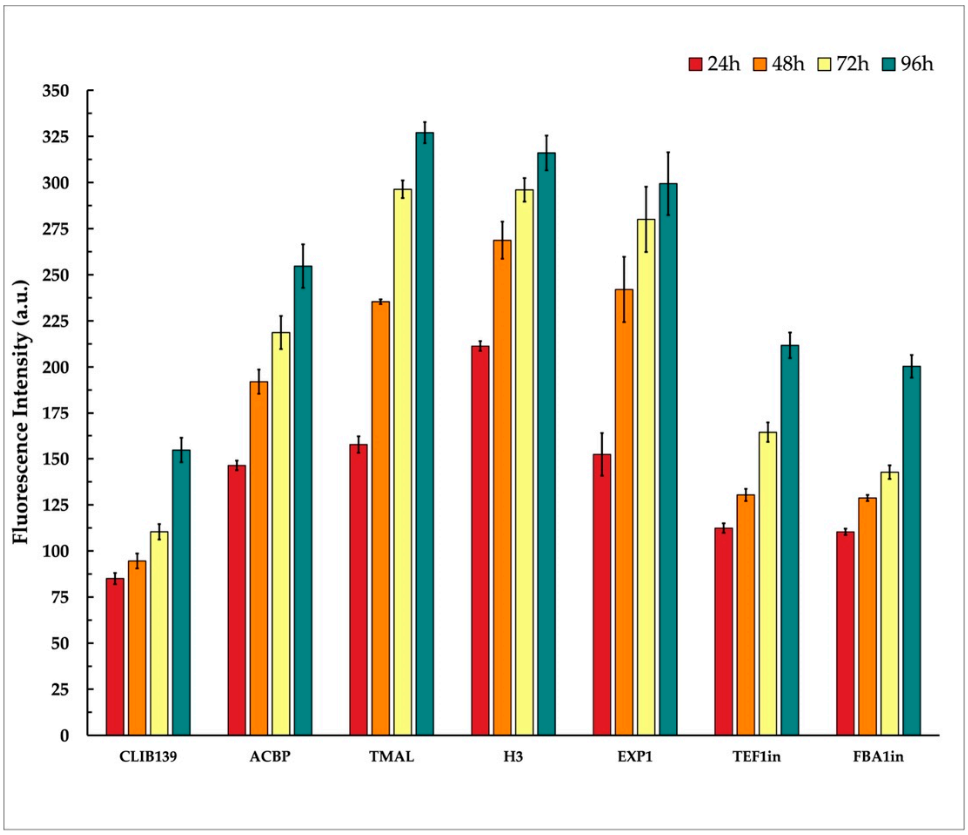

Figure S2. Promoter activity of the endogenous promoters in the *Y. lipolytica* strain Pold (CLIB139), as determined using flow cytometry and presented as fluorescence intensity of the reporter mCherry protein after cultivation in the YPG medium for 96 hours. These data were generated using the BL3-H (695/40) filter on the Attune NxT Acoustic Focusing Cytometer.

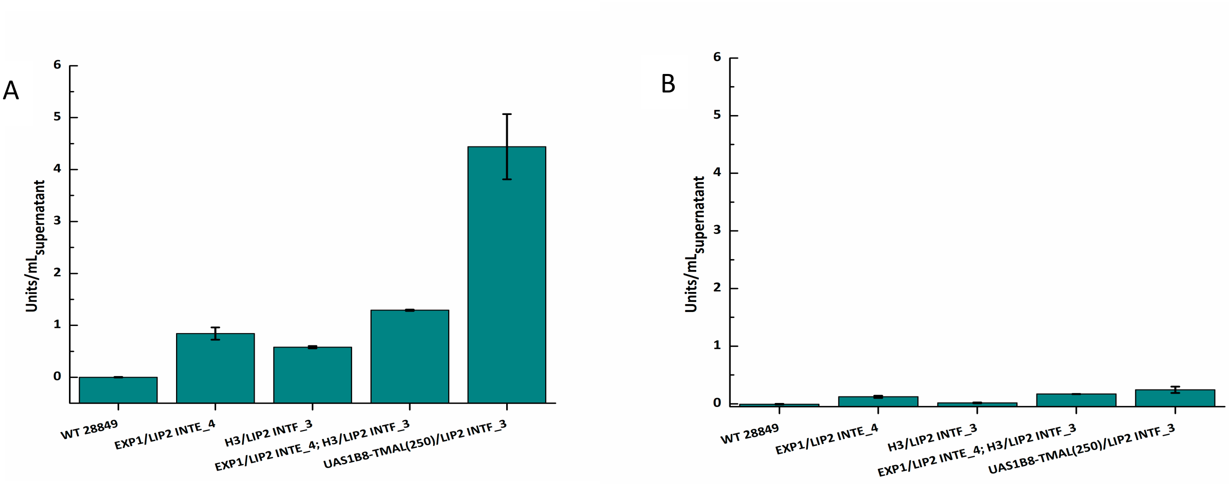

Figure S3. Effect of promoter type and *LIP2* copy number on the enzymatic activity (Units/mL$_{supernatant}$) of the recombinant *Yarrowia* strains bearing the newly constructed integrated *LIP2* expression cassetes and grown in A) YPG and B) synthetic media. Hydrolytic activity was photometrically (410nm) determined using the pNPB assay as described in Materials and Methods and expressed as the amount of enzymatic preparation (LIP2 secreted in 1 mL culture medium) that generates 1 µmol pNP per minute at 30°C (1 Unit).

Table S1. List of plasmids

Plasmid	Relevant Characteristics	Source
**Parental replicative vectors**		
pBlueScript SK-	AmpR, f1 ori, pUC ori	
pBlue-ARS18	AmpR, f1 ori, pUC ori, ARS18	This study
pBlue-ARS18-prEXP1-HYG	AmpR, f1 ori, pUC ori, ARS18, loxP, prEXP1-HphSyn-TTef, loxP	This study
pBlue-ARS18-prTEF1in-NAT	AmpR, f1 ori, pUC ori, ARS18, loxP, prTEF1intron-NAT-Tcyc1, loxP	This study
**Basic episomal vectors for promoter study**		
pHYLTEF1in	AmpR, f1 ori, pUC ori, ARS18, prTEF1intron, TSynth2, loxP, prEXP1-HphSyn-TTef, loxP	This study
pHYLEXP1	AmpR, f1 ori, pUC ori, ARS18, prEXP1, TSynth2, loxP, prEXP1-HphSyn-TTef, loxP	This study
pNYLEXP1	AmpR, f1 ori, pUC ori, ARS18, prEXP1, TSynth2, loxP, prTEF1intron-NAT-Tcyc1, loxP	This study
pNYLH3	AmpR, f1 ori, pUC ori, ARS18, prH3, TSynth2, loxP, prTEF1intron-NAT-Tcyc1, loxP	This study
pNYLACBP	AmpR, f1 ori, pUC ori, ARS18, prACBP, TSynth2, loxP, prTEF1intron-NAT-Tcyc1, loxP	This study
pNYLTMALtrim	AmpR, f1 ori, pUC ori, ARS18, prTMALtrim, TSynth2, loxP, prTEF1intron-NAT-Tcyc1, loxP	This study
pNYLFBA1in	AmpR, f1 ori, pUC ori, ARS18, prFBA1intron, TSynth2, loxP, prTEF1intron-NAT-Tcyc1, loxP	This study
pCRISPRyl	prUAS1B8-TEF(136)	Ian Wheeldon [1]
pBlueUAS1B8-TEF(136)	AmpR, f1 ori, pUC ori, prUAS1B8-TEF(136)	This study
pBlueUAS1B8-H3(260)	AmpR, f1 ori, pUC ori, prUAS1B8-H3p(260)	This study
pBlueUAS1B8-TMAL(250)	AmpR, f1 ori, pUC ori, prUAS1B8-TMALp(250)	This study
pNYLUAS1B8-TEF(136)	AmpR, f1 ori, pUC ori, ARS18, prUAS1B8-TEF(136), TSynth2, loxP, prTEF1intron-NAT-Tcyc1, loxP	This study
pNYLUAS1B8-H3(260)	AmpR, f1 ori, pUC ori, ARS18, prUAS1B8-H3(260), TSynth2, loxP, prTEF1intron-NAT-Tcyc1, loxP	This study
pNYLUAS1B8-TMAL(250)	AmpR, f1 ori, pUC ori, ARS18, prUAS1B8-TMAL(250), TSynth2, loxP, prTEF1intron-NAT-Tcyc1, loxP	This study
**Episomal vectors with *mCherry* gene**		
pNYLEXP1/mCherry	AmpR, f1 ori, pUC ori, ARS18, prEXP1-mCherry*-TSynth2, loxP, prTEF1intron-NAT-Tcyc1, loxP	This study
pNYLH3/mCherry	AmpR, f1 ori, pUC ori, ARS18, prH3-mCherry*-TSynth2, loxP, prTEF1intron-NAT-Tcyc1. loxP	This study
pNYLACBP/mCherry	AmpR, f1 ori, pUC ori, ARS18, prACBP-mCherry*-TSynth2, loxP, prTEF1intron-NAT-Tcyc1, loxP	This study
pNYLTMALtrim/mCherry	AmpR, f1 ori, pUC ori, ARS18, prTMALtrim-mCherry*-TSynth2, loxP, prTEF1intron-NAT-Tcyc1, loxP	This study
pNYLFBA1in/mCherry	AmpR, f1 ori, pUC ori, ARS18, prFBA1intron-mCherry*-TSynth2, loxP, prTEF1intron-NAT-Tcyc1, loxP	This study
pHYLTEF1in/mCherry	AmpR, f1 ori, pUC ori, ARS18, prTEF1intron-mCherry*-TSynth2, loxP, prEXP1-HphSyn-TTef, loxP	This study

pNYLUAS1B8-TEF(136)/mCherry	AmpR, f1 ori, pUC ori, ARS18, prUAS1B8-TEF(136)-mCherry*-TSynth2, loxP, prTEF1intron-NAT-Tcyc1, loxP	This study
pNYLUAS1B8-H3p(260)/mCherry	AmpR, f1 ori, pUC ori, ARS18, prUAS1B8-H3p(260)-mCherry*-TSynth2, loxP, prTEF1intron-NAT-Tcyc1, loxP	This study
pNYLUAS1B8-TMALp(250) /mCherry	AmpR, f1 ori, pUC ori, ARS18, prUAS1B8-TMALp(250)-mCherry*-TSynth2, loxP, prTEF1intron-NAT-Tcyc1, loxP	This study
**Basic EasyCloneYALI integrative vectors used**		
pCfB4785	AmpR, pUC ori, INTF_3 UP, TPex20, TLip2, loxP, prTEFintron-NAT-Tcyc1, loxP, INTF_3 DW	Irina Borodina [2]
pCfB4787	AmpR, pUC ori, INTE_4 UP, TPex20, TLip2, loxP, prTEFintron-NAT-Tcyc1, loxP, INTE_4 DW	Irina Borodina [2]
pCfB6576	AmpR, pUC ori, INTE_3 UP, TPex20, TLip2, loxP, prEXP1-HphSyn-TTef, loxP, INTE_3 DW	Irina Borodina [2]
**Basic integrative vectors for promoter study**		
pYLH3 INTF_3	AmpR, pUC ori, INTF_3 UP, TPex20, prH3, TLip2, loxP, prTEFintron-NAT-Tcyc1, loxP, INTF_3 DW	This study
pYLTMALtrim INTF_3	AmpR, pUC ori, INTF_3 UP, TPex20, prTMALtrim, TLip2, loxP, prTEFintron-NAT-Tcyc1, loxP, INTF_3 DW	This study
pYLEXP1 INTF_3	AmpR, pUC ori, INTF_3 UP, TPex20, prEXP1, TLip2, loxP, prTEFintron-NAT-Tcyc1, loxP, INTF_3 DW	This study
pYLUAS1B8-TEF(136) INTF_3	AmpR, pUC ori, INTF_3 UP, TPex20, prUAS1B8-TEF(136), TLip2, loxP, prTEFintron-NAT-Tcyc1, loxP, INTF_3 DW	This study
pYLUAS1B8-H3(260) INTF_3	AmpR, pUC ori, INTF_3 UP, TPex20, prUAS1B8-H3(260), TLip2, loxP, prTEFintron-NAT-Tcyc1, loxP, INTF_3 DW	This study
pYLUAS1B8-TMAL(250) INTF_3	AmpR, pUC ori, INTF_3 UP, TPex20, prUAS1B8-TMAL(250), TLip2, loxP, prTEFintron-NAT-Tcyc1, loxP, INTF_3 DW	This study
**Integrative vectors with mCherry gene**		
pYLH3/mCherry INTF_3	AmpR, pUC ori, INTF_3 UP, TPex20, prH3-mCherry*-TLip2, loxP, prTEFintron-NAT-Tcyc1, loxP, INTF_3 DW	This study
pYLTMALtrim/mCherry INTF_3	AmpR, pUC ori, INTF_3 UP, TPex20, prTMAL-mCherry*-TLip2, loxP, prTEFintron-NAT-Tcyc1, loxP, INTF_3 DW	This study
pYLEXP1/mCherry INTF_3	AmpR, pUC ori, INTF_3 UP, TPex20, prEXP1-mCherry*-TLip2, loxP, prTEFintron-NAT-Tcyc1, loxP, INTF_3 DW	This study
pYLUAS1B8-TEF(136)/mCherry INTF_3	AmpR, pUC ori, INTF_3 UP, TPex20, prUAS1B8-TEF(136)-mCherry*-TLip2, loxP, prTEFintron-NAT-Tcyc1, loxP, INTF_3 DW	This study
pYLUAS1B8-H3(260)/mCherry INTF_3	AmpR, pUC ori, INTF_3 UP, TPex20, prUAS1B8-H3(260)-mCherry*-TLip2, loxP, prTEFintron-NAT-Tcyc1, loxP, INTF_3 DW	This study
pYLUAS1B8-TMALp(250)/mCherry INTF_3	AmpR, pUC ori, INTF_3 UP, TPex20, prUAS1B8-TMAL(250)-mCherry*-TLip2, loxP, prTEFintron-NAT-Tcyc1, loxP, INTF_3 DW	This study
**Integrative vectors with LIP2 gene**		
pYLH3/LIP2-INTF_3	AmpR, pUC ori, INTF_3 UP, TPex20, prH3-LIP2-TLip2, loxP, prTEFintron-NAT-Tcyc1, loxP, INTF_3 DW	This study
pYLUAS1B8-H3p(260)/LIP2_INTF_3	AmpR, pUC ori, INTF_3 UP, TPex20, prUAS1B8-H3p(260)-LIP2-TLip2, loxP, prTEFintron-NAT-Tcyc1, loxP, INTF_3 DW	This study

Table S2. List of primers used in cloning and qPCR.

Primer	Name	Sequence	Used for
F1	5'-ARS18	ggatcccaatattacacccaagtag	pCRII-TOPO/YLARS18
R1	3'-ARS18	gatccagtctacactgattaattttc	
F2	GBN-ARS18-F	agctccaccgcggtggcggccgatcccaatattacacc	pBlueARS18
R2	GBN-ARS18-R	cagcccgggggatccactagtatccagtctacactgatt	
F3	GBN-pH-TEF1in-F	tcgaaggcctatgcggccaactagtagagaccgggttggcgg	pBlueARS18-prTEF1in-NAT, pH-TEF1in-Ts2
R3	GBN-Ts2-R	atatatatatatatatatatactcgagctcgaggtcgacgagctcgaatt	
F4	GBN-PrEXP1-F	atcgataccgtcgacctcgagaaggagtttggcgcccgttttttc	pBlueARS18-prEXP1-HYG
R4	GBN-TTEF-R	cactatagggcgaattgggtacaattcggacacgggcatctc	
F5	GBN-pCFB-TEF1in-F	acttcaacggaatgcgtgcgagagaccgggttggcggcgca	pYLTEF1in INTE_4
R5	GBN-pCFB-TEF1in-R	gaacagaaggaatgcacgcgctcgaggtcgacgagctcgaattcggatcccctgcggttagtactgcaaaaag	
F6	GBN-pH-EXP1-SmaI-F	gaaggcctatgcggccaactagtcccgggaaggagtttggcgcccgt	pH-EXP1-Ts2
R6	GBN-mcs-EXP1	gacgagctcgaattcggatcctgctgtagatatgtcttgtg	
F7	GBN-TEF1in-F	aatcagtgtagactggatactagtggaggcctatgcggccaac	pHTEF1in
R7	GBN-Ts2-R-YL	aacgggcgccaaactcctttcctttgaaagatgatactct	
F8	GBN-ARS-EXP1-SmaI	agtgtagactggatactagtcccgggaaggagtttggc	pNYLEXP1
R8	GBN-Ts2-Tef1-R	atgcgccgccaacccggtctctagctctttgaaagatgatactctt	
F9	FBA1p-F	tgagtgcgtacgtagcaacaacag	pCRII-TOPO/YLFBA1in
R9	FBA1-E2-R	tgtgctctcgggcgtactcgaagag	
F10	GBN-ARS18-FBA1in-F	actggatactagtcccgggccctttgagtgcgtacgtagcaacaacag	pNYLFBA1in
R10	GBN-pNYL-FBA1-E2-R	acgagctcgaattcggatccttgtgctctcgggcgtactcgaagag	
F11	YL_H3prom-F	gtgcgcgagtctgtctatgagctctct	pCRII-TOPO/YLH3
R11	YL_H3prom-R	tgtatttgtttgttggagtggttgagt	
F12	GBN-pNYL-H3p-F	agactggatactagtcccggggtgcgcgagtctgtctatg	pNYLH3
R12	GBN-pNYL-H3p-R	gtcgacgagctcgaattcggatcctgtatttgtttgttggagtg	
F13	YL-ACBPprom-F	accggtcctaagtgatacccaacga	pCRII-TOPO/YLACBP, pCRII-TOPO/ACBP(n)
R13	YL-ACBPprom-R	ggtggtaatgtgggtgttgtgtggag	pCRII-TOPO/YLACBP
F14	YALI0 C06237prom-F	cgctaagtagtacaagctacaagcac	pCRII-TOPO/YLTMAL
R14	YALI0 C06237prom-R	ggttggagtagtggtggtggggagtg	
R15	YL-ACBP-X-R	tggtggtaatgtgggtgttgtgtggagatgacgacaccttgagcggtgtatatggcgtta	pCRII-TOPO/ACBP(n)
F15	TMAL-trim-XmaI	cccgggagagagtcaatgggagagtcga	pCRII-TOPO/TMALtrim
R16	YTMAL-BamHI-R	ggatccggttggagtagtggtggtgg	
F16	GBN-pNYL-ACBPp-F	tgtagactggatactagtcccgggaccggtcctaagtgatacc	pNYLACBP(n)
R17	GBN-pNYL-ACBPp-R	cgacgagctcgaattcggatccggtggtaatgtgggtgttgtgtggag	
F17	XmaI-UAS1-F	cccgggttcgaaggtaccaaggaag	pCRII-TOPO/UAS1B8-TEF(136)
R18	TEF1-BglII-MfeI-XhoI	ctcgagcaattgagatcttgtggcgcgcctttgaatgattc	
F18	H3p-260-HindIII-F	aagcttcccaacaggcaaaatgcacc	

R19	H3p-260-BglII-MfeI-R	caattgagatcttgtatttgtttgttggagtggttg	pCRII-TOPO/H3p(260)
F19	TMALp-250-HindIII-F	aagcttctgtttgtgtccacag	pCRII-TOPO/TMALp(250)
R20	TMALp-250-BglII-MfeI-R	caattgagatctggttggagtagtggtggtgggga	
F20	GBN-pCfB-H3-F	acttcaacggaatgcgtgcggtgcgcgagtctgtctatga	pYLH3 INTF_3
R21	GBN-pCfB-H3-R	gaacagaaggaatgcacgcgatctcgaggaattcggatcctgtatttgtttgttggagt	
F21	GBN-pCfB-EXP1-F	acttcaacggaatgcgtgcgaaggagtttggcgcccgtt	pYLEXP1 INTF_3
R22	GBN-pCfB-EXP1-R	acagaaggaatgcacgcgatctcgaggtcgacgagctcgaattcggatcctgctgtagatatgtcttgtg	
F22	GBN-pCfB-TMALtr-F	acttcaacggaatgcgtgcgcccgggagagagtcaatgggaga	pYLTMALtrim INTF_3
R23	GBN-pCfB-TMALtr-R	ccgaacagaaggaatgcacgcgatctcgagctcgagatcgacgagctcgaattc	
F23	GBN-pCfB-Xma-UAS	acttcaacggaatgcgtgcgcccgggttcgaaggtaccaagg	pYLUAS1B8-TEF(136) INTF_3, pYLUAS1B8-H3(260) INTF_3, pYLUAS1B8-TMAL(250) INTF_3
R24	GBN-pCfB-mcs-R	tccgaacagaaggaatgcacgcgctcgagcaattgagatct	
F24	YlLIP2-BamHI	ggatccatgaagctttccaccatcctcttcacag	pCII-TOPO/YLLIP2
R25	YLIP2-R-XhoI	ctcgagttagataccacagacaccctcggtgacgaag	
F25	RT-YlTEF1-a-L	ccgttcttgattgccacactgccca	RT-qPCR of TEF1
R26	RT-YlTEF1-a-R	agcaacggtctgtcgcatgtctcg	
F26	RT-YlH3-L	cgaaaggtcaccggtggaaaggct	RT-qPCR of H3
R27	RT-YlH3-R	tgggcaatctctcggacaagtcgc	
F27	RT-YlACBP-L	agcttcccaagactccttccgacga	RT-qPCR of ACBP
R28	RT-YlACBP-R	tgtactcctgctcagcctcctcct	
F28	RT-YlAQP-L	acccttgtgtcactctcgccctca	RT-qPCR of AQP
R29	RT-YlAQP-R	tgggcagtgcagaacatctcgatcca	
F29	RT-YlDIOX-L	aggctacctccctcccatcctccaa	RT-qPCR of DIOX
R30	RT-YlDIOX-R	gctgctcgtcgttgatgtcctggt	
F30	RT-YALI0F00484-L	tttccagacccgggccattctcga	RT-qPCR of GK
R31	RT-YALI0F00484-R	actcggtcatgccctcggtgttga	
F31	RT-YALI0_C06237-L	atgtcccacaccaagaacgccctc	RT-qPCR of TMAL
R32	RT-YALI0_C06237-R	taaagtcgggccgctggatgttgc	

Table S3. Composition of the synthetic medium.

Component	Working Concentration (10% v/v)	Working Concentration (7.5%v/v)
Pure glycerol (ml l^{-1})	100	75
$(NH_4)_2SO_4$ (g l^{-1})	3	3
$CaCl_2$ x2H_2O (g l^{-1})	0,20	0,20
$FeCl_3$ (g l^{-1})	0,02	0,02
Thiamin-HCl (g l^{-1})	0,001	0,001
H3BO3 (g l^{-1})	0,0005	0,0005
$CuSO_4$ x 5H_2O (g l^{-1})	0,00006	0,00006

KI (g l^{-1})	0,0001	0,0001
$MnSO_4$ x H_2O (g l^{-1})	0,00045	0,00045
$ZnSO_4$ x $7H_2O$ (g l^{-1})	0,000710	0,000710
Na2MoO4 x $2H_2O$ (g l^{-1})	0,00023	0,00023
KH_2PO_4 (g l^{-1})	1	1
Na2HPO4 x 2H2O (g l^{-1})	1,3	1,3
MgSO4 x7H2O (g l^{-1})	1	1

References

1. Schwartz, C.M.; Hussain, M.S.; Blenner, M.; Wheeldon, I. Synthetic RNA Polymerase III Promoters Facilitate High-Efficiency CRISPR-Cas9-Mediated Genome Editing in Yarrowia lipolytica. *ACS Synth Biol* **2016**, *5*, 356-359, doi:10.1021/acssynbio.5b00162.
2. Holkenbrink, C.; Dam, M.I.; Kildegaard, K.R.; Beder, J.; Dahlin, J.; Doménech Belda, D.; Borodina, I. EasyCloneYALI: CRISPR/Cas9-Based Synthetic Toolbox for Engineering of the Yeast Yarrowia lipolytica. *Biotechnology Journal* **2018**, *13*, 1700543, doi:10.1002/biot.201700543.
